# Supplementary material for: Fas cell surface death receptor controls hepatic lipid metabolism by regulating mitochondrial function
Source: Nat Commun. 2017 Sep 7;8:480. doi: 10.1038/s41467-017-00566-9 (PMC5589858; doi:10.1038/s41467-017-00566-9)
Supplement: Supplementary file 1 — Supplementary Information [file 41467_2017_566_MOESM1_ESM.pdf]

File name: Supplementary Information
Description: Supplementary figures.

File name: Peer review file
Description:

Supplementary Figure 1

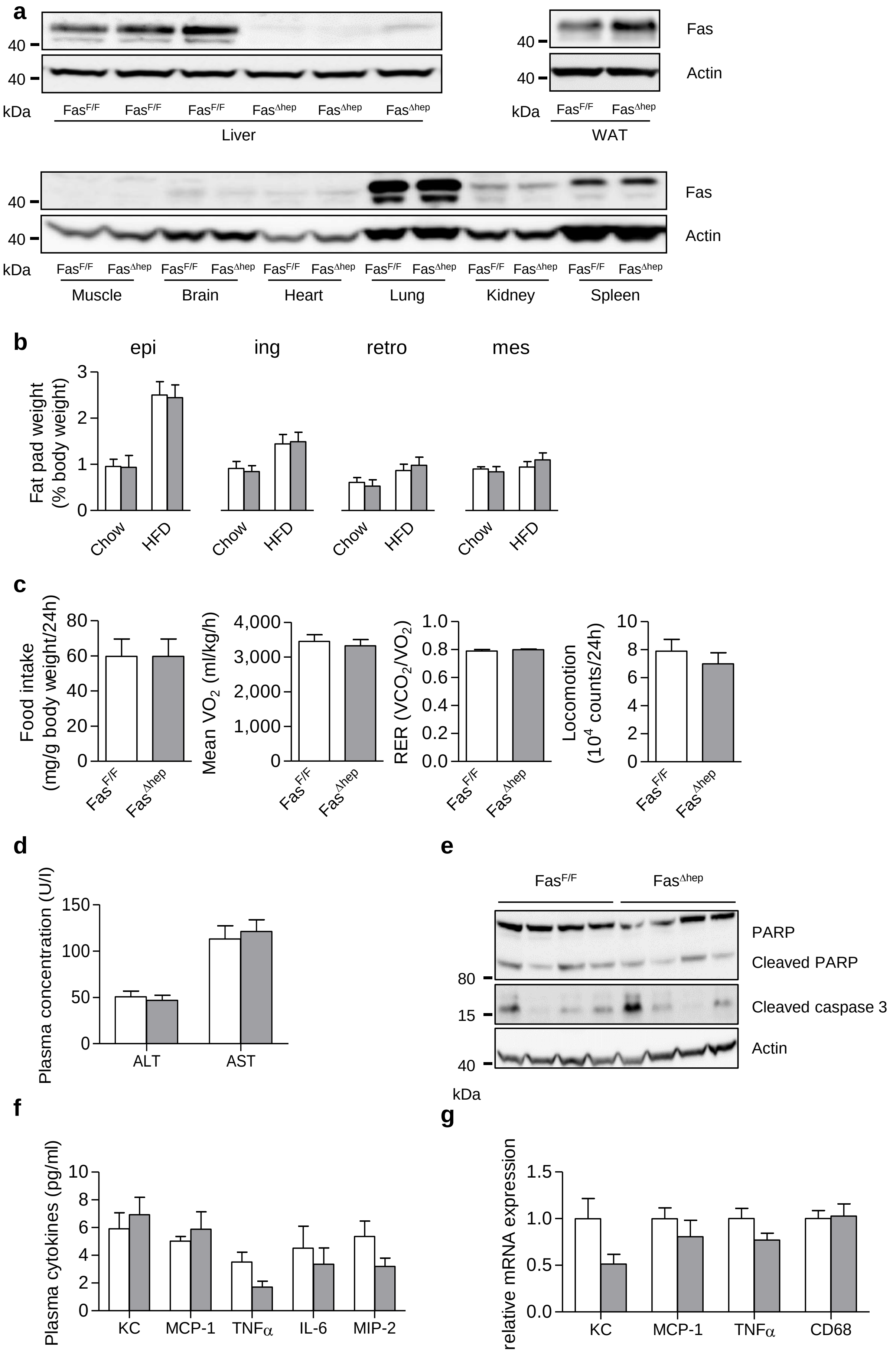

h

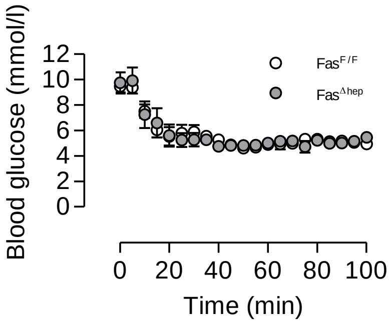
Blood glucose (mmol/l)
12
10
8
6
4
2
0
0 20 40 60 80 100
Time (min)
FasF/F
FasΔhep

i

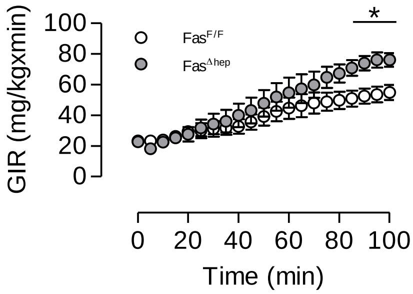
GIR (mg/kgxmin)
100
80
60
40
20
0
*
0 20 40 60 80 100
Time (min)
FasF/F
FasΔhep

j

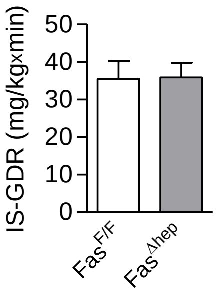
IS-GDR (mg/kgxmin)
50
40
30
20
10
0
FasF/F
FasΔhep

k

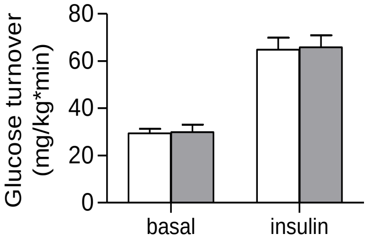
Glucose turnover
(mg/kg*min)
80
60
40
20
0
basal
insulin

l

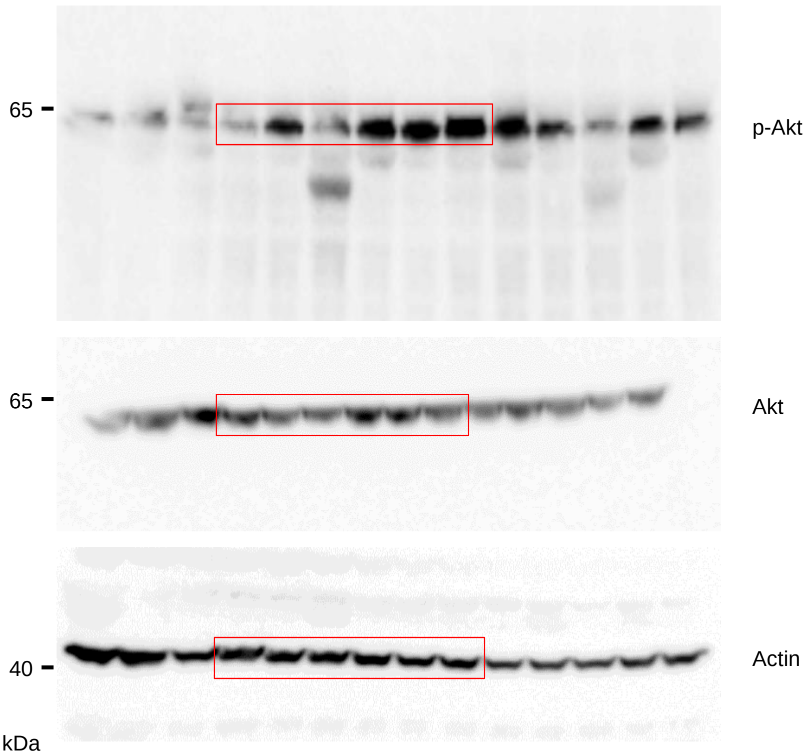
65
p-Akt
65
Akt
40
Actin
kDa

Supplementary Figure 1 Characteristics of $Fas^{F/F}$ and $Fas^{\Delta hep}$ mice

(**a**) Western blot analysis of Fas protein levels in respective tissues of $Fas^{F/F}$ and $Fas^{\Delta hep}$ mice. (**b**) Fat pad weights of chow-fed $Fas^{F/F}$ (n=3) and $Fas^{\Delta hep}$ (n=5) as well as HFD-fed $Fas^{F/F}$ (n=8) and $Fas^{\Delta hep}$ (n=6) mice. White bars: $Fas^{F/F}$ mice; grey bars: $Fas^{\Delta hep}$ mice. (**c**) Food intake, mean oxygen consumption (VO2), respiratory exchange ratio (RER) and total locomotor activity were determined in metabolic cages in HFD-fed $Fas^{F/F}$ and $Fas^{\Delta hep}$ mice (n=4). (**d**) Serum alanine aminotransferase (ALT) and aspartate aminotransferase (AST) levels of HFD-fed $Fas^{F/F}$ (white bars, n=9) and $Fas^{\Delta hep}$ (grey bars, n=8) mice. (**e**) Western blot analysis of PARP, cleaved PARP (PARP1) and caspase 3 protein levels in livers of HFD-fed $Fas^{F/F}$ and $Fas^{\Delta hep}$ mice. (**f**) Plasma cytokines of HFD-fed $Fas^{F/F}$ (white bars, n=9) and $Fas^{\Delta hep}$ (grey bars, n=8) mice and (**g**) hepatic mRNA expression of pro-inflammatory factors of HFD-fed $Fas^{F/F}$ (white bars, n=6) and $Fas^{\Delta hep}$ (grey bars, n=5) mice. (**h**) Blood glucose levels were clamped during hyperinsulinemic-euglycemic clamp at about 5 mmol/l. (**i**) In order to maintain euglycemia, glucose infusion rate was adjusted over time. Calculated insulin-stimulated glucose disposal rate (IS-GDR) after reaching a steady-state (**j**) as well as basal and steady-state glucose-turnover (**k**) are presented for $Fas^{F/F}$ (white bars; n=5) and $Fas^{\Delta hep}$ (grey bars; n=5) mice. Data are expressed as mean ± SEM. *$p<0.05$ (Student's t test). (**l**) Uncropped Western blots shown in Fig. 1g. WAT: white adipose tissue; Epi: epididymal; ing: inguinal; retro: retroperitoneal; mes: mesenteric; KC: keratinocyte-derived cytokine; MCP-1: monocyte chemoattractant protein-1; TNFα: tumor necrosis factor alpha; IL-6: interleukin-6; MIP-2: macrophage inflammatory protein-2; CD68: cluster of differentiation 68.

Supplementary Figure 2

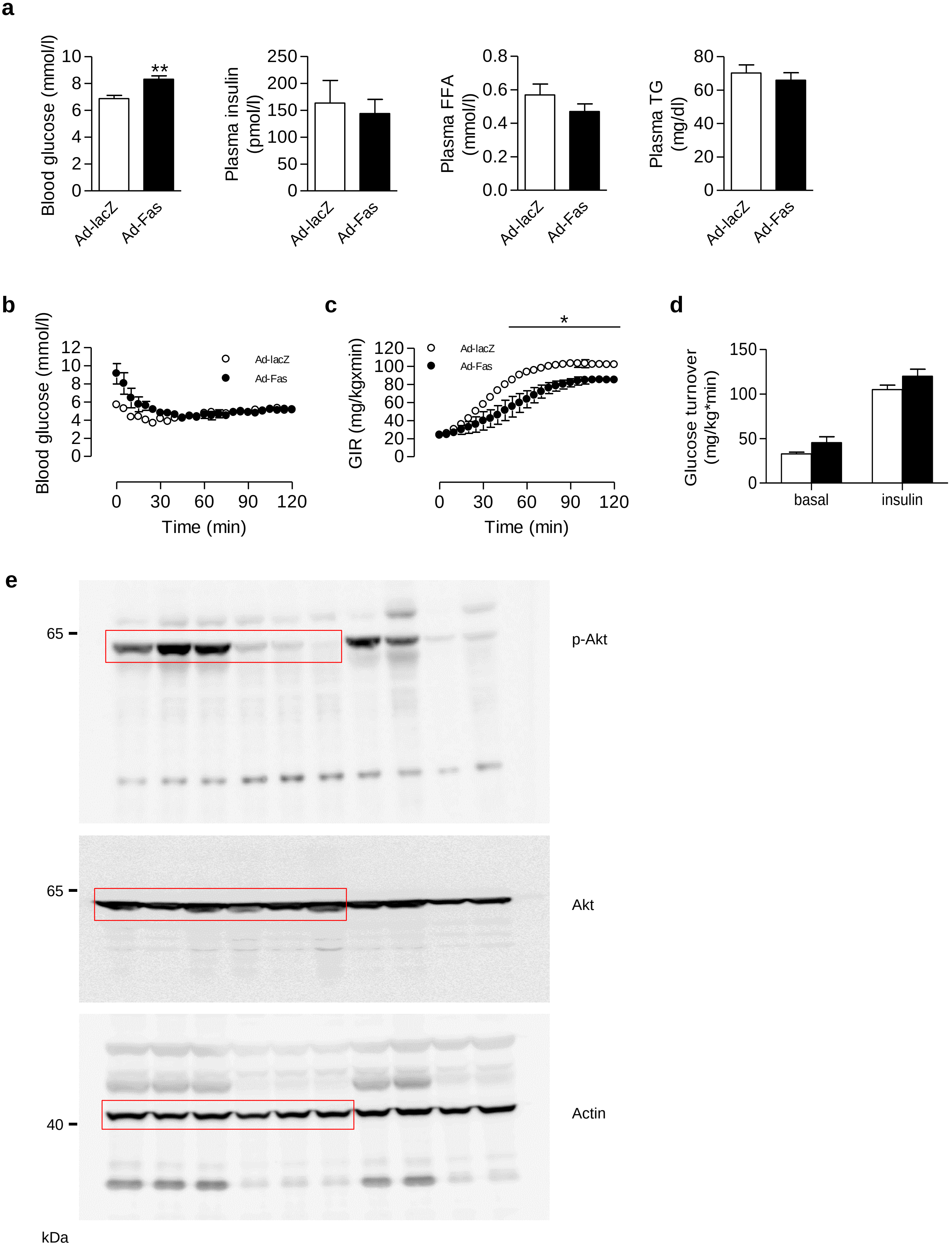

Supplementary Figure 2 Characteristics of mice injected with adenoviruses expressing lacZ or Fas

(**a**) Blood glucose, plasma insulin, FFA, and TG levels of Ad-lacZ and Ad-Fas mice (n=7). Blood glucose levels (**b**), glucose infusion rate (GIR) (**c**) as well as basal and steady-state glucose-turnover (**d**) during hyperinsulinemic-euglycemic clamp are presented for Ad-lacZ (white bars) and Ad-Fas (black bars) mice (n=4). Data are expressed as mean ± SEM. *$p<0.05$, **$p<0.01$ (Student's t test). (**e**) Uncropped Western blots shown in Figure 2f.

Supplementary Figure 3

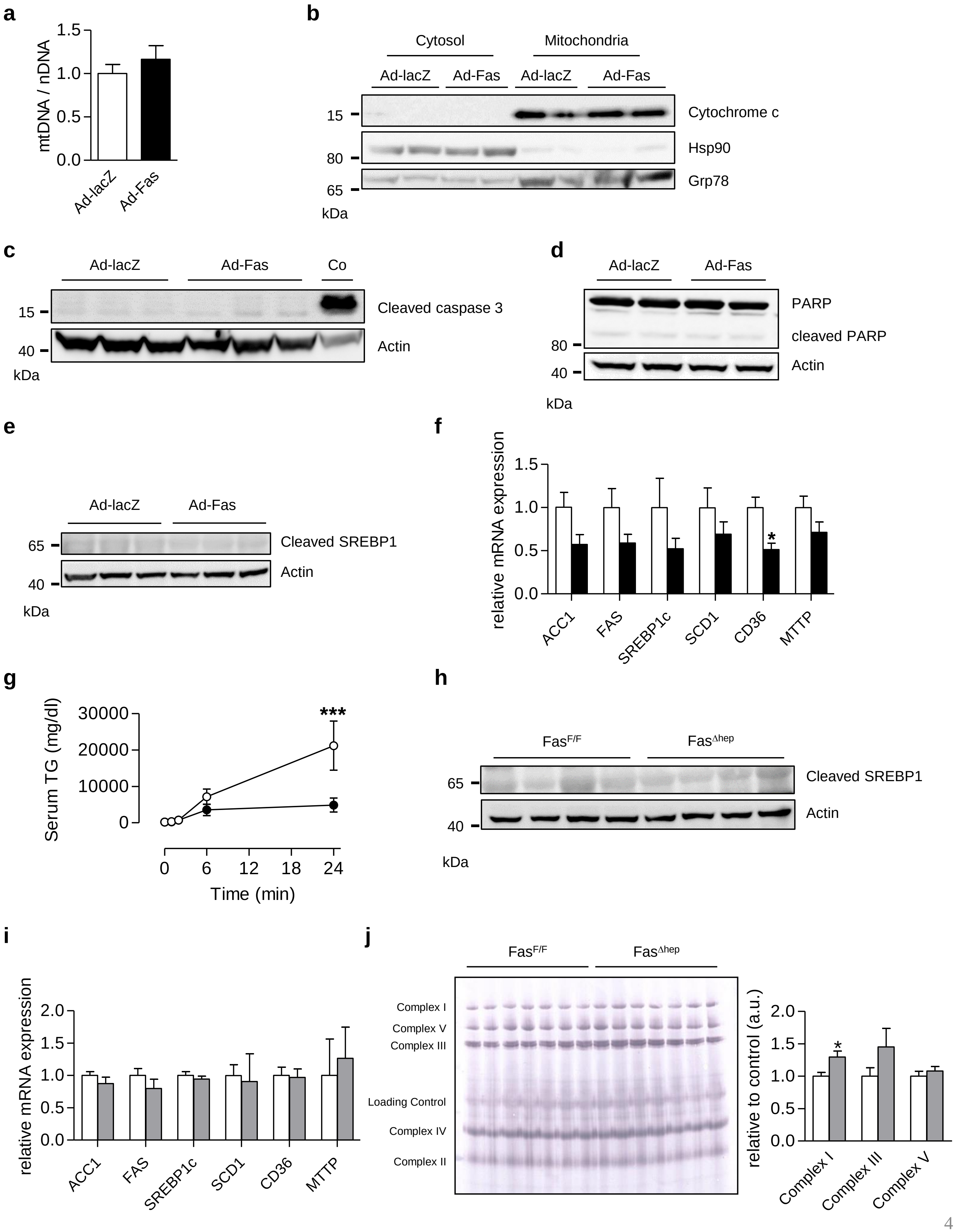

Supplementary Figure 3 Fas impacts on hepatic mitochondrial function and fatty acid oxidation

(**a**) Mitochondrial DNA abundance in liver expressed as mitochondrial DNA amount (HK2) relative to genomic DNA (Cox2) in Ad-lacZ (n=6) and Ad-Fas (n=5) mice. (**b**) Western blot analysis of cytochrome c in cytosolic and mitochondrial liver fractions. Tissue samples were harvested 5 days after virus injection. Representative Western blot of cleaved caspase 3 (**c**), PARP and cleaved PARP (**d**) or cleaved SREBP1 (**e**) in liver of Ad-lacZ or Ad-Fas mice. Tissue samples were harvested 5 days post-injection. (**f**) Hepatic mRNA expression (n=4) and (**g**) hepatic triglyceride secretion as measured by Triton-induced hypertriglyceridemia (n=7) in Ad-lacZ (white circles) and Ad-Fas (black circles) mice. (**h**) Western blot analysis of cleaved SREBP1 in liver of HFD-fed $Fas^{F/F}$ and $Fas^{\Delta hep}$ mice. (**i**) Hepatic mRNA expression in HFD-fed $Fas^{F/F}$ (white bars) and $Fas^{\Delta hep}$ (grey bars) mice (n=6). (**j**) Blue native (BN) polyacrylamide gel electrophoresis (PAGE) using isolated mitochondria from liver of HFD-fed $Fas^{F/F}$ (white bars) and $Fas^{\Delta hep}$ (grey bars) mice (n=7). A nonspecific band was used as loading control. Quantification of individual band was performed using ImageJ. Values are expressed as mean ± SEM. *$p<0.05$ (Student's t test). HK2: hexokinase 2; Cox2: cyclooxygenase-2; PARP: poly (ADP-ribose) polymerase; Co: positive control; ACC1: acetyl-CoA-carboxylase 1; FAS: fatty acid synthase; SREBP1: sterol regulatory element-binding protein 1; SCD1: stearoyl-CoA desaturase-1; CD36: cluster of differentiation 36; MTTP: microsomal triglyceride transfer protein.

Supplementary Figure 4

a

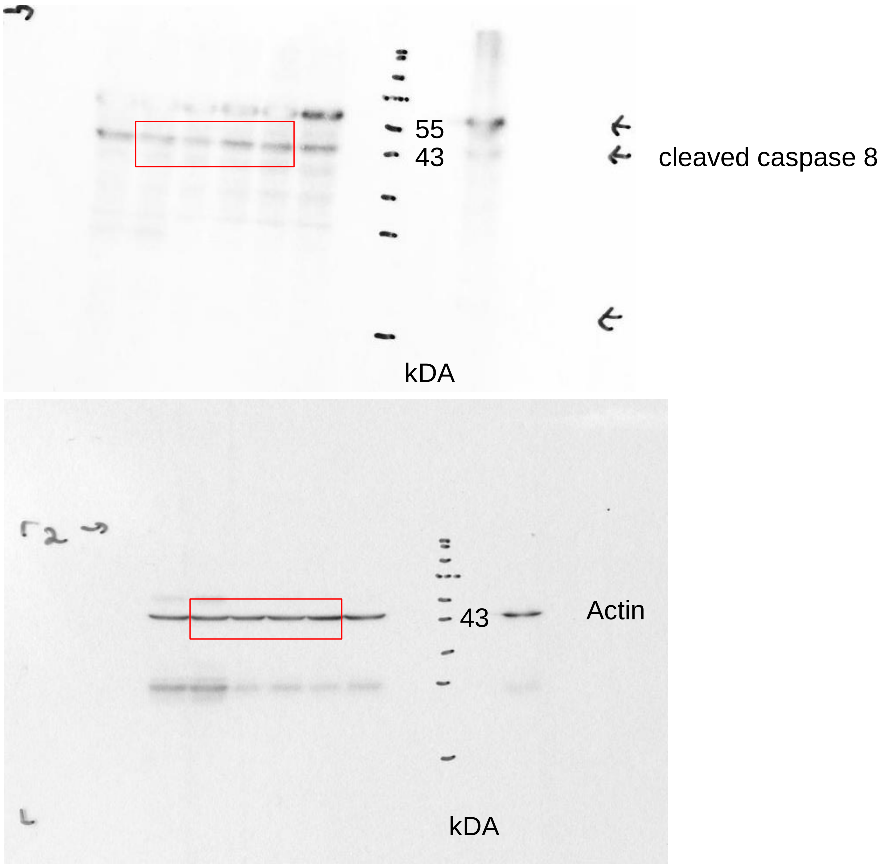

b

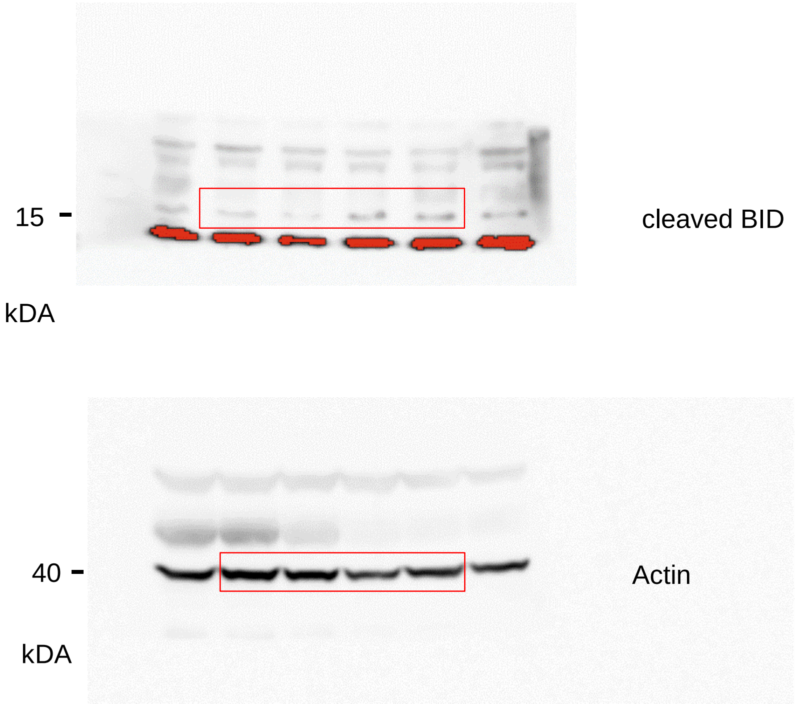

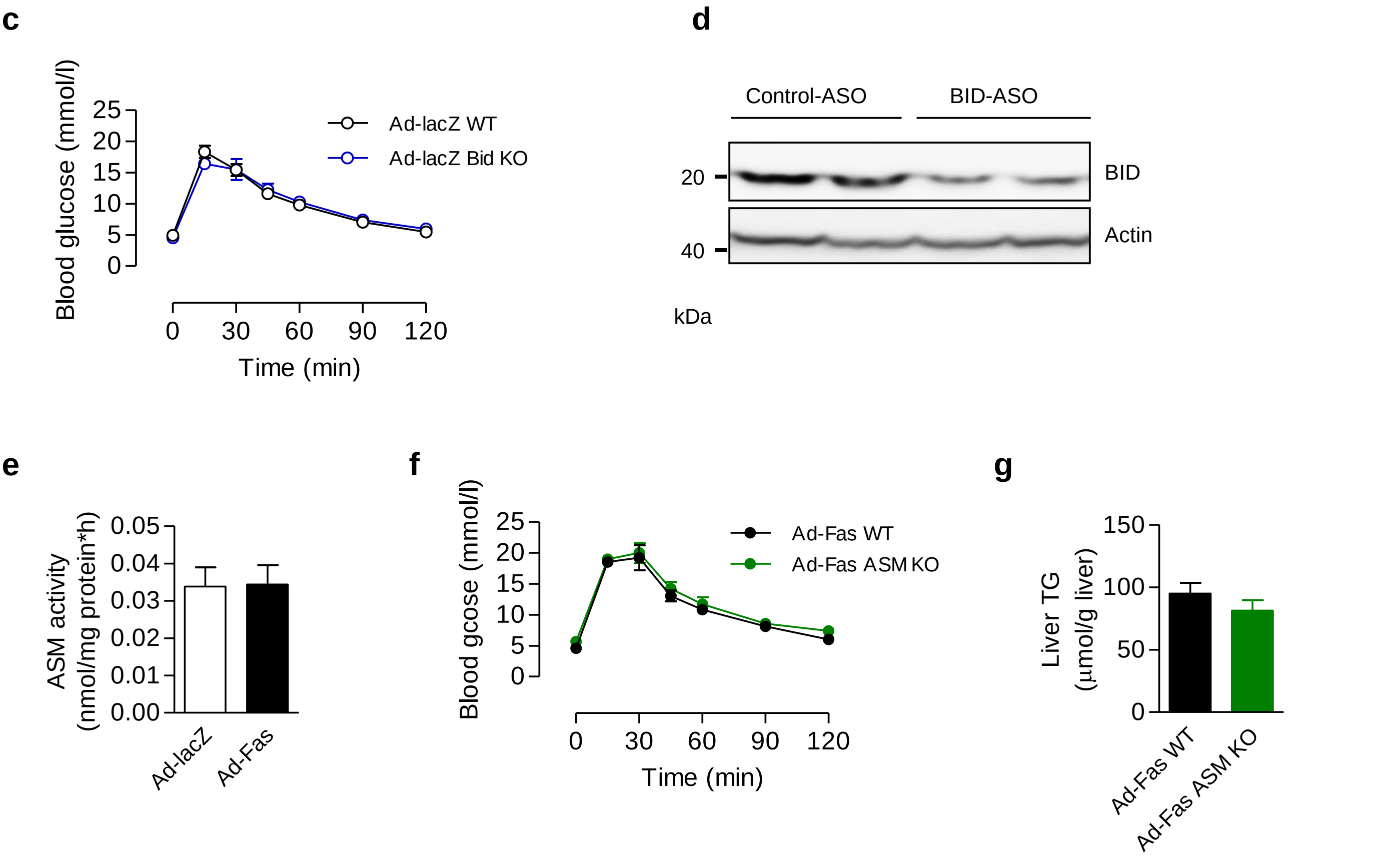

Supplementary Figure 4 Fas-activation affects glucose tolerance and liver TG levels Bid but not ASM-dependently

(**a**) and (**b**) uncropped Western blots shown in Figures 4a and 4b. (**c**) Intra-peritoneal glucose-tolerance test in WT (n=4) and Bid KO (n=6) mice 12 days after injection of Ad-lacZ is depicted. (**d**) Representative Western blot analyses of BID protein levels in liver tissue of mice treated with Control-ASO or BID-ASO. (**e**) ASM activity was determined in livers harvested from WT mice 15-16 days after injection of Ad-lacZ or Ad-Fas mice (n=7). (**f**) Intra-peritoneal glucose-tolerance test in WT (n=4) and ASM KO (n=5) mice 12 days after injection of Ad-Fas is depicted. (**g**) Liver TG levels in WT (n=4) and ASM KO (n=5) mice 16 days after injection of Ad-Fas are depicted. Data are expressed as mean ± SEM.

Supplementary Figure 5

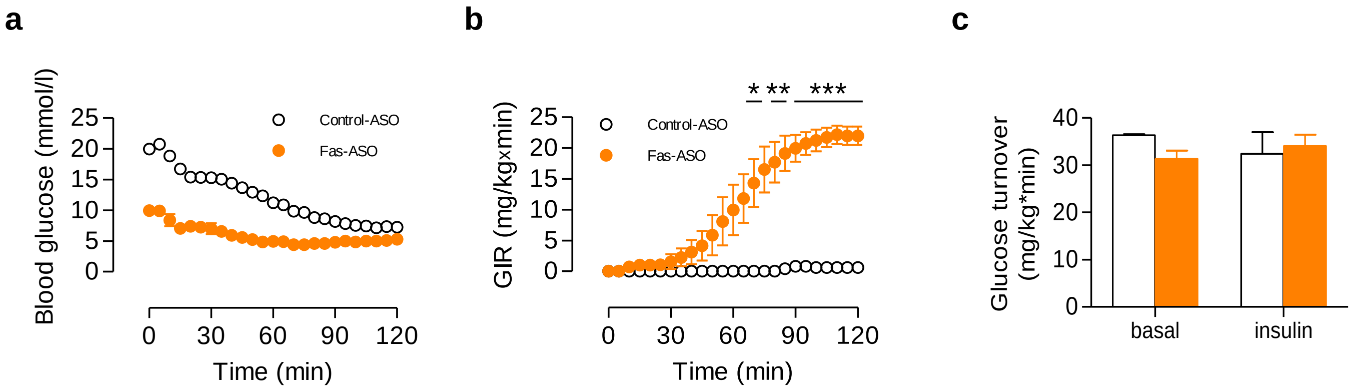

Supplementary Figure 5 Glucose infusion rate and glucose turnover in mice treated with Fas antisense oligonucleotides (ASO)

(**a**) Blood glucose levels were clamped during hyperinsulinemic-euglycemic clamp at approx. 5 mmol/l. (**b**) In order to maintain euglycemia, glucose infusion rate was adjusted over time. (**c**) Basal and steady-state glucose-turnover is presented. Control-ASO (white bars; n=4) or Fas-ASO (orange bars; n=5) injected mice. Data are expressed as mean ± SEM. *p<0.05, **p<0.01, ***p<0.001 (Student's t test).

Supplementary Figure 6

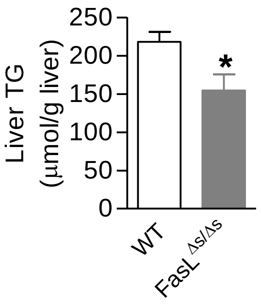

Supplementary Figure 6 Reduced hepatic steatosis in HFD-fed mice deficient of secreted FasL

Liver triglyceride levels in HFD-fed WT (n=7) and FasL$^{\Delta s/\Delta s}$ (n=6) mice are depicted. Data are expressed as mean ± SEM. *p<0.05 (Student's t test).
